# Plant–plant communication in *Camellia japonica* and *C. rusticana* via volatiles

**DOI:** 10.1038/s41598-024-56268-y

**Published:** 2024-03-15

**Authors:** Yusuke Sakurai, Satomi Ishizaki

**Affiliations:** Graduate School of Science and Technology, Niigata University, Niigata, 950-2181 Japan

**Keywords:** Plant ecology, Evolutionary ecology

## Abstract

Plants emit volatile compounds when they are subjected to herbivorous, pathogenic, or artificial damages. Both the damaged plant and the neighboring intact plants induce resistance when they receive these volatiles, a phenomenon known as plant–plant communication. However, field observations of this phenomenon are limited. To understand the nature of plant–plant communication, we collected information about intra- and inter-plant signaling via volatiles in *Camellia japonica* and *C. rusticana* under natural conditions. We exposed intact branches of damaged plant (intra-plant) or neighboring plant (inter-plant) to artificially damaged plant volatiles (ADPVs). Leaf damage reduced in ADPVs-exposed branches in the neighboring plants compared to branches that were exposed to volatiles from intact leaves, thus, indicating that inter-plant signaling occur by the emission of volatiles from damaged leaves. We also conducted an air-transfer experiment wherein the headspace air of the damaged branch was transferred to the headspace of intact branches. Leaf damage reduced on the ADPVs-transferred branch compared to the control branch. The effect of volatiles on damage reduction lasted for three months. Our results indicate that ADPVs in *Camellia* species contain cues that induce resistance in neighboring plants. Our findings improve understanding of plant defense strategies that may be used in horticulture and agriculture.

## Introduction

In natural environments, plants attacked by insects and other herbivores respond by changing various traits to reduce damage (i.e., resistance) and/or maintain their fitness (i.e., compensation). One of these responses is induced resistance, which is the ability of a plant to increase its resistance to herbivores after being damaged^1^. In some cases, resistance is induced not only in the damaged plant but also in its neighbors, especially when it is induced by volatiles released from the damaged site. This phenomenon is referred to “plant–plant communication.” Volatiles increase and/or prime resistance in neighboring plants^2^. For instance, exposure to (*Z*)-3-hexenyl acetate can induce floral nectar secretion in lima bean plants^3^. *Arabidopsis thaliana* seedlings treated with (*E*)-2-hexenal induce the transcription of several genes including lipoxygenase and phenylalanine ammonia lyase genes, which are involved in plant defense responses^4^. In tea plants, after exposure to indole, one of the major herbivore-induced plant volatiles, the plant hormone salicylic acid, which regulates defensive responses to pathogens, is induced, while gibberellic acid and indole-3-acetic acid concentrations, which regulate growth, are reduced^5^.

Plants emit volatiles from damaged parts because volatiles can act as signaling cues to induce resistance in damaged and neighboring plants^6^. In plants, systemic resistance is generally induced by internal cues that move primarily through vascular traces from the damaged site^7^. However, the movement of internal cues can be restricted by the degree of vascular connectivity between the plant parts^8,9^. For instance, damaged leaves without vascular connections have a weaker induction of resistance than those with vascular connections^10^. However, mobile herbivores, such as caterpillars and grasshoppers, can move among branches regardless of the vascular connections in the plants. Therefore, limiting the movement of internal cues will cause herbivore evasion and induce resistance. However, if the cues are volatile, they diffuse without any limitation by vascular connections. Indeed, external signaling via volatiles is particularly important among branches of woody plants and shrubs, whose branches are believed to share limited vascular connectivity and compensate for restricted internal signaling^11–13^.

Moreover, some plants use volatiles for signaling between individuals. Plants can respond to the volatiles emitted by damaged neighbors but not to those of intact neighbors. Resistance in neighbors is induced because natural enemies are attracted to the surroundings of the volatile source^14–17^, or because neighboring plants eavesdrop on the volatiles from damaged plants^18^. Contrastingly, plants select information on volatiles and respond only to adaptively valuable information. For example, in a high-risk herbivory environment, *Solidago altissima* evolved to respond to volatiles from damaged neighbors, regardless of whether they had kinship^19^, while in low-risk environments, it responded only to volatiles from genetically identical plants.

More than 39 species can respond to volatiles from damaged neighbors^20^, including woody and shrub species, such as *Alnus glutinosa*^21^*, Artemisia tridentata*^22^, *Salix exigua*^23^, *S. lemmonii*^23^, *S. eriocarpa*^24^, *Populus tremula* × *tremuloides*^25^, and *Fagus crenata*^26^, and herbaceous species, such as *Phaseolus lunatus*^3^*, Solidago altissima*^19,27^, *Sorghum bicolor*^28^*, Brassica oleracea*^29^, and *B. nigra*^29^, which includes communication in root systems ^30^. However, most research on plant–plant communication has been conducted in laboratories, and examples of field experiments are few^21–23,31^. To understand the importance of plant–plant communication in ecosystems and the evolutionary factors (e.g., growth environment, phylogeny, and life history) under which plant–plant communication occurs, field tests are necessary. Camellia tree is a member of the tea genus containing various distinct chemical compounds in its leaves. It is a common tree grown on roadside in Japan. Herein, we examined the presence or absence of volatile-mediated intra- and plant–plant communication in *Camellia japonica* and *C. rusticana* in the field.

## Results

**Figure 1:**
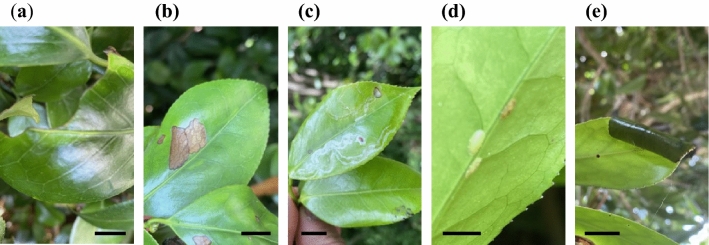
Type of damage observed on *Camellia* leaves: (**a**) chewing, (**b**) disease, (**c**) leaf mining, (**d**) coccid, and (**e**) leaf roll. Scale bars indicate 10 mm (**a**–**c**, **e**), or 5 mm (**d**).

Five types of damages were observed in the field: chewing, disease, leaf mining, coccids, and leaf roll (Fig. 1). Although we did not identify all the herbivorous or pathogenic species that caused damage, *Euproctis pseudoconspersa* (Lepidoptera; Lymantriidae), which chewed leaves, was identified. Two experiments were conducted as described below.

### Experiment 1. Field experiment for communication by volatiles

**Figure 2:**
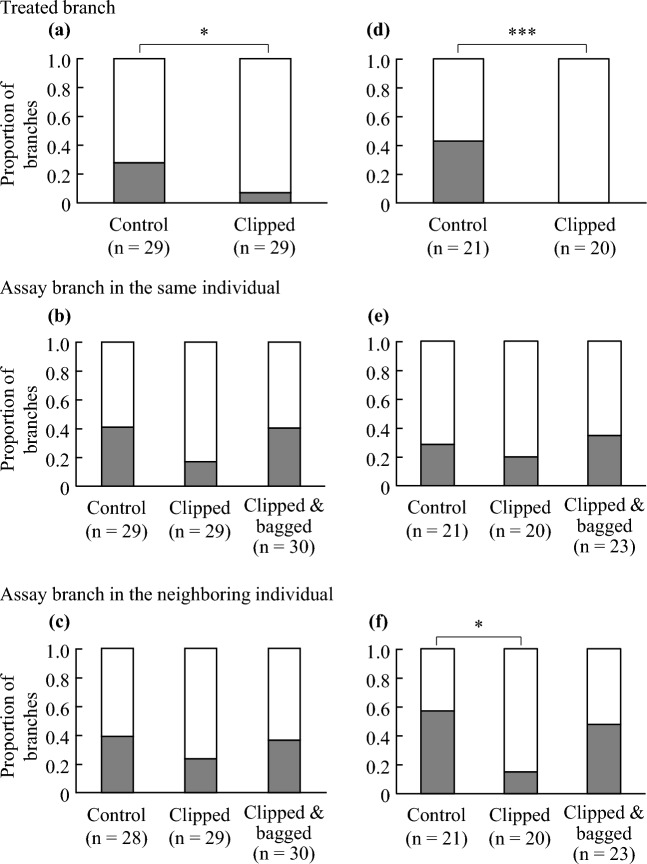
Proportion of damaged and undamaged branches in Experiment 1 conducted in 2020 (**a**–**c**) and 2021 (**d**–**f**). (**a**, **d**) Treated branch, (**b**, **e**) assay branch in the same plant, and (**c**, **f**) assay branch in the neighboring plant. Filled and open bars indicate damaged and undamaged branch, respectively. n indicates the number of branches in each treatment. Asterisks indicate the result of statistics (analysis of deviance for treated branch, Dunnett’s multiple comparison for the assay branch in the same and neighboring plant): *P < 0.05; ***P < 0.001.

**Figure 3:**
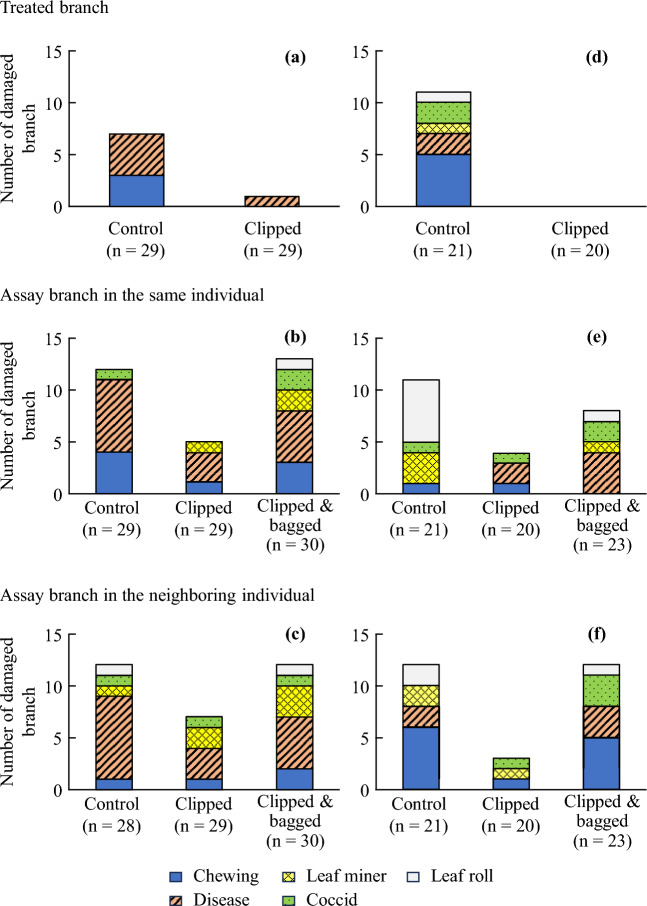
Number of branches damaged by different types of herbivores in Experiment 1 in 2020 (**a**–**c**) and 2021 (**d**–**f**). (**a**, **d**) Treated branch, (**b**, **e**) assay branch in the same plant, and (**c**, **f**) assay branch in the neighboring plant. Branches that suffered multiple damage types were counted in each damage type.

In the first experiment, the effect of treatment was significant in the treated branch but not in the assay branch of the same and neighboring individuals in 2020 (Table 1; Fig. 2a–c), while in 2021 the effect of treatment was significant in treated branch and assay branch in the neighboring individuals (Table 1; Fig. 2d–f). *Camellia* species and branch position relative to treated branch (branch connection) were not significantly affected on proportion of damaged individuals (Tables S1, S2), while the effect of interactions between species and treatment was significant on the branch in the same plants in 2020. Although the interaction between species and treatment was partially significant, we pooled data regardless plant species and branch connection in order to gain sample size for each branch type. Regardless of the statistical significance, the clipped treatment reduced the proportion of damaged individuals in all three branch types, whereas the clipped and bagged treatments did not (Fig. 2). In terms of the damage type, chewing and disease damage were the most common, both of which were reduced by the clipped treatment (Fig. 3).

**Table 1:** Analysis of deviance of GLM with binomial distribution fitted on proportion of damaged plant for field experiment in 2020 and 2021.

Year	Source	Treated branch			Assay branch in the same individual			Assay branch in the neighboring individual		
		LR χ^2^	Df	*P*	LR χ^2^	Df	*P*	LR χ^2^	Df	*P*
2020	Treatment	4.84	1	0.028*	5.60	2	0.061	2.01	2	0.366
2021	Treatment	14.47	1	< 0.001***	1.19	2	0.553	9.03	2	0.011*

*P < 0.05; **P < 0.01; ***P < 0.001.

### Experiment 2. Volatile transfer experiment

**Figure 4:**
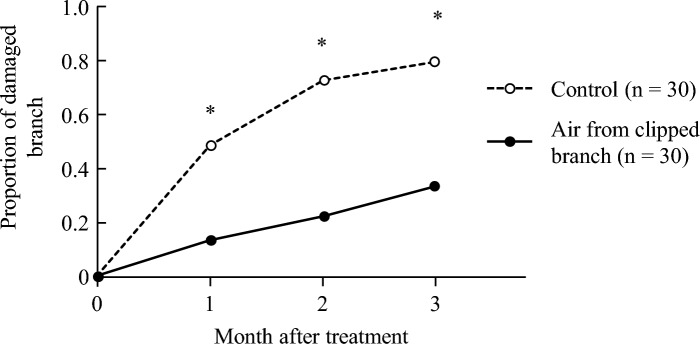
Proportion of damaged branches in Experiment 2. Asterisk indicates significant difference between treatments (Fisher’s exact test followed by Holm’s correction, adjusted *P* < 0.05).

There was less damage in the branches exposed to air from the clipped treatment compared to the control treatment for three months (Fisher’s exact test for 1st month, adjusted *P* = 0.005; for 2nd months, adjusted *P* < 0.001; for 3rd months, adjusted *P* < 0.001, Fig. 4). The damage types included chewing, disease, leaf mining, coccids, and leaf roll.

## Discussion

Plants can induce systemic resistance following local damage. To induce systemic resistance, internal or airborne cues are transmitted from damaged to intact plant parts. When airborne cues are released, they carry information about herbivorous attacks not only to the damaged plant but also to neighboring plants^20,22,32^.

In the present study, field experiments were conducted to test the ability of *C. japonica* and *C. rusticana* to communicate with plants using volatiles. Experiment 1 showed that volatiles emitted from damaged leaves induced resistance in neighboring plants. Although the effect of treatment on the assay branch of the neighboring plants was not statistically significant in the first year, the tendency was the same as the following year. Damage was reduced only by the clipped treatment on the assay branch in the neighboring plant (Figs. 2c,f and 3c,f), indicating that the volatiles emitted from damaged leaves influenced the resistance level in neighboring individuals. However, in our first experiment, the possibility that volatiles attracted natural enemies or that herbivores avoided volatiles, instead of communication, could not be rejected because volatiles could diffuse into the air surrounding the assay branch^33–35^. Therefore, in Experiment 2, we placed the assay branches away from the air-donor branches. Damage to the assay branch in the same individual was not significantly affected by treatments, although clip treatment tended to reduce damage (Fig. 2b,e). These results will indicate that other branch of damaged plant could not induce resistance. However, other factors may also have influenced; responses of plants to treatments would have been different between species in 2020, or other environmental factors may have differed among plants.

In Experiment 2, resistance was induced when the branches were exposed to volatiles from damaged leaves (Fig. 4). Air was collected from the surroundings of the donor plant and transferred to the assay plant, which was grown away from the donors. Therefore, the assay branches may not have been protected by attracting natural enemies or repelling herbivores. Moreover, the effect of volatiles persisted for at least three months after treatment (Fig. 4). These results suggested that the volatiles released from excised *C. japonica* and *C. rusticana* leaves contained cues that induced systemic resistance. The tea plant *C. sinensis* is known to emit indole when attacked by caterpillars^5^, which primes the expression of early defense genes and the production of jasmonates and defense-related secondary metabolites in neighboring tea plants^5^. Similar to tea plants, our results indicated that *C. japonica* and *C. rusticana* would release cues from damaged leaves.

Sagebrush, which grows in North America, and goldenrod, which is native to North America and a serious invasive species in Japan, are known to induce stronger resistance to volatiles released by closely related individuals or clones than to those released by distantly related individuals^19,31,36^. The use of volatiles allows information to be transmitted quickly and spatially to nearby closely related individuals.

Among the damage types, chewing and disease were reduced in both experiments (Fig. 3), suggesting that resistance against these two types of damages appeared to be induced in the leaves in which volatile cues were detected. Resistances to insects and diseases are believed to compete, with the former depending on jasmonic acid, while the latter depending on salicylic acid^37^. Contrastingly, volatiles can induce resistance against multiple enemies. For example, indole increases the biosynthesis of salicylic acid and primes jasmonic acid biosynthesis in tea plants^5^. Mint volatiles expressed multiple resistances in neighboring soybean plants, which reduced the common cutworm (*Spodoptera litura*, Lepidoptera: Noctuidae) larval weight, number of eggs laid by spider mites (*Tetranychus urticae*, Arachnida: Trombidiformes), and the infection area of *Phakopsora pachyrhizi*^38^.

Herein, we assumed that artificial clipping of leaves caused herbivorous damage. However, leaves damaged by natural herbivores and pathogens may emit volatiles that differ from those damaged by artificial clipping in both timing and quantity. Herbivorous damage often causes volatile emissions over a longer time than artificial clipping. Natural damage by herbivores and pathogens may induce resistance more continuously and more strongly^39^. For plant–plant communication under natural conditions, the receiver plant must be near the emitter plant^40^. In our first experiment, the volatile-emitting and volatile-receiving branches were located 30 cm apart. This distance was similar to that found between branches in natural settings.

The effect of volatile exposure lasted for long time. We measured the damage one month after treatment in Experiment 1, whereas every month until three months after treatment in Experiment 2. In Experiment 2, the difference between treatments had already occurred after one month, with maximum difference at two months later (Fig. 4). Similar to our results, the effect of volatile exposure sustained for long time in sagebrush^22^. Sagebrush that was exposed volatiles in spring when newly leaves spread had less damage even in autumn^22^. In our study, we treated the newly expanding leaves in spring. Although phenology of herbivores will influence on the damage accumulation, induction of resistance from early in the season can suppress the increase of herbivore densities and accumulation of damage throughout the growing season. Although we only measured the damage one month later after treatment in Experiment 1, according the result of Experiment 2, the period of measurement after treatment had not been long or short.

We revealed that volatiles from the damaged leaves of *C. japonica* and *C. rusticana* transmitted information about their enemies and caused their resistance. The impact of information transmitted by volatiles may vary in plants that grow in different environments, such as under the high pressure of herbivore attack or in low-resource habitats. The ecological importance of plant–plant communication in the natural habitats of *C. japonica* and *C. rusticana* should be revealed by further field experiments. Moreover, the key compounds that transmit information of damage and the expression pattern of defense-related genes after volatile exposure remain unclear. Once these molecular biological perspectives are clarified, it will be possible to elucidate the evolutionary process of plant–plant communication and protect trees from enemies via volatiles in forests or agricultural fields.

## Materials and methods

### Plant materials

*Camellia japonica* L. and *C. rusticana* Honda (Theaceae) were grown at the Niigata University Ikarashi campus (Niigata, Japan; 37°52′ N, 138°56′ E). Both species are evergreen shrubs. *Camellia japonica* is widely distributed throughout Japan, except in Hokkaido, mainly along the Pacific coast, where snow fall during winters is low. It grows in dense forests throughout the year, reaches a height of 10–15 m, and has a tree topology with an erected main stem^41^. *Camellia rusticana* is distributed at an elevation of ca. 300–1400 m on the Sea of Japan side in the Tohoku and Hokuriku regions, which experience heavy snowfall during winter. It has adapted to heavy snowfall through creeping branches and thin cuticular layers^42^. At the study site, both species flower in April and May, when the snow melts. It took approximately two months for the branches and leaves to grow, and the leaves that expanded two years before fell off when new leaves started growing in spring. In fall, when the surrounding trees were defoliated, the tree received more sun light. In October, the shoot opened and dropped seeds on the forest floor. Seeds germinate around May; however, individuals born from seeds are extremely rare.

### Experiment 1. Field experiment for communication by volatiles

In total, 90 pairs of adjacent individuals were selected for this study. Paired plants were selected randomly throughout our study site. These were *C. japonica* planted on the roadside and *C. rusticana* planted in the deciduous forest. In 2020, we used both species in our experiments, although the same *Camellia* species were selected in each pair (64 pairs of *C. japonica* and 26 pairs of *C. rusticana*). In 2021, we used only *C. japonica*. Our study site was not in the natural distribution area of *C. rusticana*; however, there was a garden where *C. rusticana* was planted.

**Figure 5:**
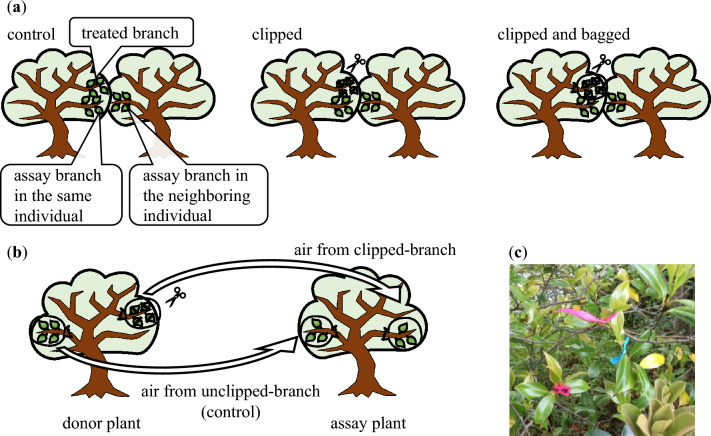
(**a**) Schematic diagram of each branch and treatment in Experiment 1. For each pair, three branches were selected; two from one individual, with one assigned as “treated branch” and the other as “assay branch in the same individual,” and one branch from another individual assigned as “assay branch in the neighboring individual”. The treated branches of each pair were subjected to one of the three treatments: (i) control, (ii) clipped wherein half of the leaves on the treated branches were cut with scissors, and (iii) clipped and bagged wherein half of the leaves on the treated branches were cut with scissors and then bagged with plastic bags to inhibit the diffusion of volatiles. (**b**) Schematic diagram of each branch and treatment in Experiment 2. Two branches of assay plant were used; one receiving air from the headspace of an experimentally clipped air-donor branch, and the other receiving air from the headspace of an unclipped control branch. Head space air of donor branch was transferred to the branch head space of assay plant using a large 500-mL plastic syringe. (**c**) Photograph of branches in Experiment 1. Newly grown branches were used. Branch with pink-numbered tape (lower left) was treated branch; branch with pink tape (upper left) was assay branch in the same individual; branch with blue tape (upper right) was assay branch in the neighboring individual.

For each pair, we selected three branches; two from one individual, with one assigned as “treated branch” and the other as “assay branch in the same individual,” and one branch from another individual was assigned as “assay branch in the neighboring individual” (Fig. 5a). All selected branches were newly grown in the current year (current-year branches) and immediately after leafing out. Additionally, the assayed branches of both the same and neighboring individuals were located within 30 cm of the treated branch. In 2020, we also recorded the branch connection between treated branch and assay branch in the same individuals; adjacent branch (*n* = 10), branching from the same lateral branch (*n* = 53), connecting via trunk (*n* = 26). Because the branch connection was not affected damage (Table S2), we selected the branch which connected to treated branch via trunk as assay branch in the same individuals in 2021. The treated branches of each pair were subjected to one of the following three treatments: (i) control wherein only numbering was applied to the treated branches, (ii) clipped wherein half of the leaves on the treated branches were cut with scissors so that the volatiles diffused from the excised leaves to the surrounding area, and (iii) clipped and bagged wherein half of the leaves on the treated branches were cut with scissors and then bagged with plastic bags to inhibit the diffusion of volatiles. Each plastic bag was sealed with a binding band. The treatments were applied on May 22, 2020, and May 13, 2021.

1 month later (June 19, 2020, and June 22, 2021), we measured whether there was any damage, such as feeding damage or diseases, to the treated branch, assay branch in the same individual, and assay branch in the neighboring individual. The response of damaged branches was measured in the treated branches. If the damage was reduced compared to the control, it indicated that resistance was induced. We did not measure the damage to the treated branches of the clipped and bagged treatment because the bags were maintained throughout the experimental period. Sample sizes decreased due to missing of several samples in 2020. In addition, a hedge of *Camellia* which was planted near our study site was cut down during the experiment in 2021. Cutting the hedge would have resulted in an unintended release of volatiles. Therefore, individuals near that hedge were deleted from analysis.

### Experiment 2. Volatile transfer experiment

To confirm that the volatiles released from damaged leaves included signals that induced resistance, we experimentally transferred headspace air from the treated branches to the assay branch (Fig. 5b). The methods described previously^43^ were used after modifications. Thirty *Camellia* plants were selected from the Niigata University campus, and two branches with three young leaves were marked on each tree. These branches were used as assay branches and assigned to one of the two treatments: one receiving air from the headspace of an experimentally clipped air-donor branch, and the other receiving air from the headspace of an unclipped control branch. Air-donor plants were placed approximately 5 m from the assay plants. Several branches of each air-donor plant were covered with new plastic bags on May 22, 2021. On half of these branches, the leaves were cut off before enclosing them in a plastic bag and used as the air-donor source. The other branches served as control branches and were not clipped. After clipping, the plastic bag surrounding each branch was sealed with a binding band. Volatiles were collected from the clipped branches in plastic bags for 24 h.

After 24 h, a small hole was drilled into the side of the plastic bag surrounding the air donor. Air was drawn through the hole from the headspace of each donor branch with a large 500-mL plastic syringe (Antianyucheng, Beijing, China) and then moved to the corresponding assay branch. Before injecting the air, the assay branches were covered with a new plastic bag tied with the binding band. Air from the syringe was injected into the plastic bag surrounding the assay branch through a small hole on the side of the bag. After air injection, the small hole was tied with a bandage. After 24 h, the bags were removed, and after treatment, the presence or absence of damage to the assay branches and the types of damage were recorded every month for three months (June 22, July 22, and August 22, 2021).

### Statistical analysis

All statistical analyses were performed using R ver. 4.0.3^44^. Generalized linear model (GLM) with binominal distribution and analysis of deviance were used to analyze the effect of species, branch connection and treatment on the proportion of damaged individuals in Experiment 1. All GLMs were constructed with damage (damaged or intact) as response variable. To analyze species and its interaction between treatment, species (2 levels) and treatment (2 levels for treated branch, 3 levels for assay branch in the same and neighboring individuals) were included to GLMs as explanatory variables. The effect of branch connection (3 levels) and its interaction with treatment were analyzed for the assay branch in the same individuals. To analyze the effect of treatment, GLMs included treatment as explanatory variables. Following the construction of GLM, comparison to control was conducted by Dunnett’s multiple comparison for the assay branch in the same and neighboring individuals. Following functions in R were used; *glm* (with binominal distribution and logit link) in MASS package for construction of GLM models, *Anova* (with likelihood ration test and type II calculation) in car package for analysis of deviance, and *glht* (with Dunnett test) in multcomp package for multiple comparisons. Experiments 2 was analyzed using Fisher’s exact probability test. The proportion of assay branches with damaged leaves were compared between clipped and control treatments using Fisher’s exact test followed by Holm’s correction (over all α = 0.05).

### Supplementary Information

Supplementary Tables.

## Data Availability

The datasets generated and/or analyzed during the current study are available in the figshare repository with https://figshare.com/s/dd740d12b65cdbc1a4f6.
