# Supplementary material for: Plant–plant communication in Camellia japonica and C. rusticana via volatiles
Source: Sci Rep. 2024 Mar 15;14:6284. doi: 10.1038/s41598-024-56268-y (PMC10943193; doi:10.1038/s41598-024-56268-y)
Supplement: Supplementary file 1 — Supplementary Tables. [file 41598_2024_56268_MOESM1_ESM.pdf]

Plant-plant communication in *Camellia japonica* and *C. rusticana* via volatiles

Yusuke Sakurai and Satomi Ishizaki

Supplementary information

Table S1 Analysis of deviance of GLM with binomial distribution fitted on proportion of damaged plants for field experiment in 2020.

	Treated branch				Assay branch in the same individual				Assay branch in the neighboring individual		
Source	LR χ^2	Df	*P*		LR χ^2	Df	*P*		LR χ^2	Df	*P*
Species	0.024	1	0.877		1.735	1	0.188		1.24	1	0.269
Treatment	4.86	1	0.028	*	5.79	2	0.055		1.94	2	0.379
Species x Treatment	0.390	1	0.532		6.432	2	0.040	*	4.73	2	0.094

*, $P < 0.05$

Table S2 Analysis of deviance of GLM with binomial distribution fitted on proportion of damaged plants for field experiment in 2020

	Assay branch in the same individual		
Source	LR χ^2	Df	*P*
Branch connection	2.13	2	0.345
Treatment	4.44	2	0.109
Branch connection x Treatment	6.61	3	0.085
